# Effect of enteral nutrition nursing combined with gastrointestinal function monitoring on gastric retention and abdominal distension in neurosurgical patients

**DOI:** 10.1097/MD.0000000000050864

**Published:** 2026-10-02

**Authors:** Huihui Lei, Man Wang, Ping Ke

**Affiliations:** a Department of Neurosurgery, Chinese PLA General Hospital of Central Theater Command, Wuhan, Hubei, China.

**Keywords:** abdominal distension, enteral nutrition, gastric retention, gastrointestinal function monitoring, neurosurgery, nursing intervention

## Abstract

This study evaluated the clinical effects of enteral nutrition nursing combined with gastrointestinal function monitoring in neurosurgical patients receiving enteral nutrition. This single-center retrospective cohort study included 120 neurosurgical patients who received enteral nutritional support from June 2023 to June 2025. Sixty patients received routine enteral nutrition nursing (control group), and 60 received routine care plus dynamic gastrointestinal function monitoring (combined nursing group). Outcomes included gastric retention, abdominal distension, nausea and vomiting, overall gastrointestinal intolerance, enteral nutrition interruption, aspiration/reflux, nutritional delivery, gastrointestinal recovery, hospital and intensive care unit (ICU) stay, nutritional tolerance, and nursing satisfaction. Logistic regression was used to identify factors associated with gastric retention. Baseline characteristics were comparable between groups (all *P* > .05). Gastric retention occurred less frequently in the combined nursing group than in the control group (15.0% vs 30.0%), although the difference did not reach statistical significance (*P* = .053). The combined nursing group had lower incidences of abdominal distension (16.7% vs 36.7%, *P* = .011), nausea and vomiting (11.7% vs 26.7%, *P* = .038), and overall gastrointestinal intolerance (20.0% vs 43.3%, *P* = .006). Time to achievement of enteral nutrition targets, gastric residual volume, time to recovery of bowel sounds, and time to first defecation were also more favorable (all *P* < .001). Enteral nutrition interruption was less frequent (8.3% vs 23.3%, *P* = .022), and hospital and ICU stays were shorter (both *P* < .01). Good nutritional tolerance and nursing satisfaction were more frequent in the combined nursing group (both *P* < .05). Multivariable analysis showed that the combined nursing model was associated with lower odds of gastric retention. Enteral nutrition nursing combined with gastrointestinal function monitoring was associated with improved gastrointestinal tolerance, more efficient nutritional delivery, earlier gastrointestinal recovery, and shorter hospital and ICU stays. A lower observed incidence of gastric retention was also noted, and adjusted analysis suggested an association with reduced odds of gastric retention.

## 1. Introduction

Neurosurgical patients frequently enter a hypercatabolic state as a consequence of traumatic brain injury, intracerebral hemorrhage, and postoperative stress responses, resulting in markedly increased energy expenditure and a heightened susceptibility to malnutrition and negative nitrogen balance, both of which may adversely affect neurological recovery and overall prognosis.^[1,2]^ Previous studies have demonstrated that poor nutritional status is closely associated with a higher risk of infection, prolonged hospitalization, and increased mortality, underscoring the clinical importance of early and standardized nutritional support in the management of critically ill neurosurgical patients.^[3]^

Among the available nutritional support modalities, enteral nutrition is generally regarded as the preferred strategy for critically ill patients because it is more physiologically compatible, helps preserve intestinal mucosal barrier integrity, and may reduce bacterial translocation as well as infection-related complications.^[1,4]^ Several international guidelines further recommend that enteral nutrition should be initiated as early as feasible once hemodynamic stability has been achieved and gastrointestinal function is deemed adequate, so as to improve overall clinical outcomes.^[2]^ Nevertheless, the implementation of enteral nutrition in neurosurgical populations remains particularly challenging.

Neurological injury may disrupt regulation along the brain–gut axis, thereby impairing gastrointestinal motility and delaying gastric emptying, which in turn substantially increases the likelihood of gastric retention and abdominal distension.^[5,6]^ Prior evidence has shown that patients with severe traumatic brain injury often experience prolonged gastric emptying time and suppressed gastrointestinal peristalsis, both of which compromise tolerance to enteral feeding.^[7]^ In addition, neurosurgical patients are frequently exposed to factors such as sedation, mechanical ventilation, and prolonged immobilization, all of which may further aggravate gastrointestinal dysfunction.^[8]^

Gastric retention and abdominal distension are among the most common complications encountered during enteral nutrition and represent major contributors to feeding intolerance and interruption of nutritional delivery.^[9,10]^ Norton et al reported that patients with brain injury often exhibit limited tolerance to enteral nutrition, with delayed gastric emptying being a prominent manifestation.^[9]^ Subsequent investigations have further confirmed that gastric retention is closely related to an elevated risk of aspiration and may negatively influence patient outcomes.^[11]^

In routine clinical practice, gastric residual volume monitoring has long been used as an indicator of gastrointestinal tolerance; however, its clinical utility has become increasingly controversial in recent years.^[11,12]^ Some studies have suggested that excessive reliance on gastric residual volume may lead to unnecessary interruption of nutritional support.^[12]^ A meta-analysis by Wang et al indicated that reducing the frequency of gastric residual volume monitoring did not increase adverse outcomes and, instead, might improve the efficiency of nutritional delivery.^[12]^

Accordingly, a single parameter is unlikely to capture gastrointestinal functional status comprehensively. Current research has therefore placed greater emphasis on multidimensional assessment strategies incorporating abdominal signs, nausea and vomiting, bowel movement patterns, and other relevant clinical indicators.^[13]^ A systematic review by Li et al suggested that such multidomain evaluation may improve the accuracy of identifying feeding intolerance.^[13]^ Nursing intervention plays a pivotal role in the delivery and management of enteral nutrition. Through continuous observation and individualized adjustment of feeding strategies, nursing care may improve gastrointestinal tolerance and reduce the occurrence of nutrition-related complications.^[10,14]^ Rhoney et al demonstrated that variations in feeding methods and nursing approaches can significantly affect nutritional tolerance in patients with brain injury.^[14]^ In addition, studies focusing on aspiration risk have suggested that appropriate gastrointestinal assessment combined with targeted nursing measures may enhance the safety of enteral nutrition.^[15]^ Therefore, integrating gastrointestinal function monitoring with nursing intervention may represent a promising approach for optimizing nutritional management in neurosurgical patients.

Against this background, the present single-center retrospective cohort study was conducted to investigate the effect of enteral nutrition nursing combined with gastrointestinal function monitoring on the incidence of gastric retention and abdominal distension in neurosurgical patients, with the aim of providing evidence to inform clinical nursing practice.

## 2. Methods

### 2.1. Study design

This study was approved by the Ethics Committee of CHINESE PLA GENERAL HOSPITAL OF CENTRAL THEATER COMMAND. This study was designed as a single-center retrospective cohort investigation. Electronic medical records, nursing documentation, enteral nutrition implementation records, and gastrointestinal function monitoring data of hospitalized neurosurgical patients treated in our institution between June 2023 and June 2025 were retrospectively reviewed to analyze the occurrence of gastrointestinal function-related complications under different nursing intervention models. In accordance with the methodological principles of retrospective research, efforts were made to minimize selection bias and information bias in order to enhance the robustness and clinical interpretability of the findings. The study protocol was approved by the hospital ethics committee. As this was a retrospective analysis based exclusively on preexisting clinical data, with no additional intervention imposed on patients, and all data were anonymized prior to analysis, the requirement for informed consent was waived.

### 2.2. Study population

A total of 120 patients who were admitted to the Department of Neurosurgery of our hospital from June 2023 to June 2025 and received enteral nutritional support were included. Patients were classified according to the nursing management documented in their medical and nursing records: 60 received routine enteral nutrition nursing (control group), and 60 received routine care plus dynamic gastrointestinal function monitoring (combined nursing group). Because this was a retrospective, non-randomized study, group allocation was based on the nursing care actually received rather than random assignment. Therefore, potential temporal, ward- or team-related, and clinical-selection bias cannot be completely excluded and was taken into account when interpreting the findings.

### 2.3. Inclusion and exclusion criteria

The inclusion criteria were as follows: fulfillment of the diagnostic criteria for neurosurgical diseases; receipt of enteral nutritional support during hospitalization for at least 3 days; age ≥ 18 years; and complete clinical and nursing records. The exclusion criteria were as follows: coexisting severe organic gastrointestinal disease; a prior history of marked gastrointestinal motility disorder or surgery affecting gastric emptying; death or discharge against medical advice shortly after admission; long-term complete dependence on parenteral nutrition; severe multiple organ failure or end-stage disease; and incomplete medical data.

### 2.4. Nursing interventions

Patients in the control group received routine enteral nutrition nursing care, including establishment and maintenance of the enteral feeding route, gradual adjustment of infusion rate and dose, elevation of the head of the bed to 30°–45°, reinforcement of tube management, and regular observation of gastrointestinal reactions. On the basis of these measures, patients in the observation group received enteral nutrition nursing combined with gastrointestinal function monitoring. Specifically, gastric residual volume, abdominal signs, and gastrointestinal tolerance were dynamically evaluated, and the enteral nutrition regimen was adjusted in a timely manner according to patient status. For patients showing a tendency toward gastric retention or abdominal distension, individualized interventions were implemented, such as reducing the infusion rate, adopting intermittent feeding, applying gastrointestinal decompression, and administering prokinetic therapy, thereby enabling continuous, dynamic, and refined nursing management.

### 2.5. Outcome measures

The study endpoints included gastric retention, abdominal distension, nausea and vomiting, overall gastrointestinal intolerance, interruption of enteral nutrition, aspiration or reflux, nutrition-related indicators, gastrointestinal functional recovery, hospitalization-related outcomes, nutritional tolerance, and nursing satisfaction. Gastric retention was identified according to the institutional enteral nutrition management protocol on the basis of an increased gastric residual volume in combination with relevant clinical and nursing documentation. Abdominal distension was identified according to documented abdominal signs and nursing records, whereas nausea and vomiting were recorded according to documented clinical manifestations. Overall gastrointestinal intolerance was defined as the occurrence of one or more clinically documented gastrointestinal manifestations that adversely affected the tolerance or continuation of enteral nutrition, including gastric retention, abdominal distension, nausea, or vomiting. Time to achievement of enteral nutrition targets, gastric residual volume, time to recovery of bowel sounds, and time to first defecation were recorded as indicators of nutritional delivery and gastrointestinal functional recovery. Nutritional tolerance was evaluated retrospectively according to the patient’s documented tolerance of enteral feeding, including gastrointestinal symptoms, feeding continuity, and the ability to achieve the prescribed enteral nutrition regimen. Patients without clinically relevant feeding intolerance requiring substantial reduction or interruption of enteral nutrition were classified as having good nutritional tolerance. Nursing satisfaction was assessed according to the routine satisfaction assessment documented in the nursing records at the end of the hospitalization. Because the nutritional tolerance classification and nursing satisfaction assessment were based on institutional clinical documentation rather than externally validated research instruments, these measures were regarded as locally defined outcomes and were interpreted with appropriate caution.

### 2.6. Data collection

Data were retrospectively extracted by trained investigators using a standardized data collection form. General patient characteristics, clinical variables, nursing records, and outcome measures were collected and independently cross-checked by 2 researchers to ensure data accuracy and completeness.

### 2.7. Statistical analysis

All statistical analyses were performed using Statistical Package for the Social Sciences version 22.0. Continuous variables with a normal distribution were expressed as mean ± standard deviation and compared between groups using the independent-samples *t* test. Non-normally distributed data were analyzed using the rank-sum test. Categorical variables were presented as frequencies and percentages and compared using the χ^2^ test or Fisher exact test, as appropriate. Logistic regression analysis was further conducted to identify factors associated with the occurrence of gastric retention and abdominal distension. A two-sided *P* value < .05 was considered statistically significant.

## 3. Results

### 3.1. Comparison of baseline characteristics

No statistically significant differences were observed between the 2 groups with respect to age, sex distribution, body mass index, underlying comorbidities (including hypertension and diabetes), the proportion of patients receiving mechanical ventilation, or Glasgow Coma Scale (GCS) score at admission (all *P* > .05), indicating good baseline comparability. As shown in Table 1, no significant between-group differences were identified in key variables such as age (58.6 ± 12.4 vs 57.9 ± 11.8 years, *t* = 0.31, *P* = .758), the proportion of male patients (60.0% vs 63.3%, χ^2^ = 0.13, *P* = .718), or the rate of mechanical ventilation (36.7% vs 40.0%, χ^2^ = 0.14, *P* = .706). In addition, the GCS scores were comparable between the control and observation groups (9.2 ± 2.1 vs 9.0 ± 2.3, *P* = .624), further suggesting a similar neurological status at admission and providing a sound basis for subsequent comparison of intervention-related effects (Table 1).

**Table 1:** Comparison of baseline characteristics.

Characteristic	Control group (n = 60)	Observation group (n = 60)	*t*/χ^2^	*P*
Age (yrs, x̄±s)	58.6 ± 12.4	57.9 ± 11.8	0.31	.758
Male, n (%)	36 (60.0)	38 (63.3)	0.13	.718
BMI (kg/m^2^)	23.4 ± 3.1	23.7 ± 3.3	0.52	.604
Hypertension, n (%)	28 (46.7)	30 (50.0)	0.13	.722
Diabetes mellitus, n (%)	15 (25.0)	17 (28.3)	0.16	.686
Mechanical ventilation, n (%)	22 (36.7)	24 (40.0)	0.14	.706
GCS score	9.2 ± 2.1	9.0 ± 2.3	0.49	.624

BMI = body mass index, GCS = Glasgow Coma Scale.

### 3.2. Comparison of primary outcome measures

With respect to the major clinical outcomes, the observation group exhibited a generally lower incidence of gastrointestinal function-related complications than the control group. As presented in Table 2, the incidence of gastric retention in the observation group was 15.0%, compared with 30.0% in the control group. Although the difference approached but did not reach conventional statistical significance (χ^2^ = 3.75, *P* = .053), a clear downward trend was evident. The incidence of abdominal distension was significantly lower in the observation group than in the control group (16.7% vs 36.7%, χ^2^ = 6.43, *P* = .011), suggesting that the combined intervention exerted a substantial beneficial effect on gastrointestinal motility. Likewise, nausea and vomiting occurred less frequently in the observation group (11.7%) than in the control group (26.7%, χ^2^ = 4.32, *P* = .038). When assessed as a composite indicator, the overall incidence of gastrointestinal intolerance was also significantly reduced in the observation group (20.0% vs 43.3%, χ^2^ = 7.68, *P* = .006), indicating that enteral nutrition nursing combined with gastrointestinal function monitoring effectively enhanced tolerance to enteral feeding (Table 2).

**Table 2:** Comparison of primary outcome measures.

Outcome	Control group (n = 60)	Observation group (n = 60)	χ^2^	*P*
Gastric retention, n (%)	18 (30.0)	9 (15.0)	3.75	.053
Abdominal distension, n (%)	22 (36.7)	10 (16.7)	6.43	.011
Nausea and vomiting, n (%)	16 (26.7)	7 (11.7)	4.32	.038
Gastrointestinal intolerance, n (%)	26 (43.3)	12 (20.0)	7.68	.006

### 3.3. Comparison of nutrition-related indicators

In terms of nutrition support-related parameters, patients in the combined nursing group demonstrated more favorable observed outcomes. As shown in Table 3, the time required to achieve the enteral nutrition target was shorter in the combined nursing group than in the control group (4.2 ± 1.5 days vs 5.8 ± 1.9 days, *t* = 5.07, *P* < .001). Gastric residual volume was lower in the combined nursing group (120 ± 50 mL vs 180 ± 65 mL, *t* = 5.63, *P* < .001). In addition, time to recovery of bowel sounds (2.8 ± 1.0 days) and time to first defecation (3.5 ± 1.2 days) were shorter than in the control group (3.9 ± 1.2 days and 4.6 ± 1.5 days, respectively; both *P* < .001). These between-group differences were associated with the combined nursing strategy but should not be interpreted as proof of a causal effect (Table 3).

**Table 3:** Comparison of nutrition-related indicators.

Parameter	Control group	Observation group	*t*	*P*
Time to target achievement (d)	5.8 ± 1.9	4.2 ± 1.5	5.07	<.001
Gastric residual volume (mL)	180 ± 65	120 ± 50	5.63	<.001
Time to recovery of bowel sounds (d)	3.9 ± 1.2	2.8 ± 1.0	5.43	<.001
Time to recovery of defecation (d)	4.6 ± 1.5	3.5 ± 1.2	4.45	<.001

### 3.4. Comparison of complications and safety outcomes

Regarding safety-related endpoints, certain differences in complication rates were observed between the 2 groups. As summarized in Table 4, the incidence of aspiration was lower in the observation group than in the control group (3.3% vs 10.0%); however, the between-group difference did not reach statistical significance (χ^2^ = 2.10, *P* = .147). A similar decreasing tendency was found for reflux (6.7% vs 16.7%, *P* = .088). No statistically significant difference was identified in the incidence of diarrhea between the 2 groups (*P* = .128). Notably, the interruption rate of enteral nutrition was significantly lower in the observation group than in the control group (8.3% vs 23.3%, χ^2^ = 5.27, *P* = .022), suggesting that the combined intervention substantially improved the continuity and stability of enteral nutrition delivery and thereby reduced the risk of treatment interruption (Table 4).

**Table 4:** Complications and safety outcomes.

Complication	Control group (n = 60)	Observation group (n = 60)	χ^2^	*P*
Aspiration, n (%)	6 (10.0)	2 (3.3)	2.1	.147
Reflux, n (%)	10 (16.7)	4 (6.7)	2.91	.088
Diarrhea, n (%)	12 (20.0)	6 (10.0)	2.31	.128
Interruption of enteral nutrition, n (%)	14 (23.3)	5 (8.3)	5.27	.022

### 3.5. Comparison of hospitalization and recovery outcomes

With regard to hospitalization-related outcomes, the combined nursing group had a shorter observed length of hospital stay than the control group (15.3 ± 4.6 days vs 18.6 ± 5.2 days, *t* = 3.67, *P* < .001). Intensive care unit (ICU) stay was also shorter in the combined nursing group (6.9 ± 2.8 days vs 8.4 ± 3.1 days, *t* = 2.79, *P* = .006; Table 5). Given the retrospective observational design, these differences should be interpreted as associations; shortened hospitalization cannot be attributed solely to the nursing model because unmeasured disease severity, complications, treatment decisions, and other residual confounders may have contributed (Table 5).

**Table 5:** Comparison of hospitalization and recovery outcomes.

Parameter	Control group	Observation group	*t*	*P*
Length of hospital stay (d)	18.6 ± 5.2	15.3 ± 4.6	3.67	<.001
ICU stay (d)	8.4 ± 3.1	6.9 ± 2.8	2.79	.006

ICU = intensive care unit.

### 3.6. Comparison of nutritional tolerance and nursing satisfaction

As presented in Table 6, 80.0% of patients in the combined nursing group were classified as having good nutritional tolerance, compared with 56.7% in the control group (χ^2^ = 7.73, *P* = .005). Nursing satisfaction was 86.7% in the combined nursing group and 66.7% in the control group (χ^2^ = 6.40, *P* = .011). The operational definitions, scoring methods, cutoffs, and assessment timing for these outcomes are specified in Section 2.5. If locally developed rather than validated instruments were used, these results should be interpreted with caution.

**Table 6:** Comparison of nutritional tolerance and nursing satisfaction.

Outcome	Control group (n = 60)	Observation group (n = 60)	χ^2^	*P*
Good tolerance, n (%)	34 (56.7)	48 (80.0)	7.73	.005
Nursing satisfaction, n (%)	40 (66.7)	52 (86.7)	6.4	.011

### 3.7. Logistic regression analysis

To further examine factors associated with gastric retention, multivariable logistic regression was performed (Table 7). Mechanical ventilation was associated with higher odds of gastric retention (odds ratio [OR] = 2.27, *P* = .047), and routine nursing was likewise associated with higher odds (OR = 2.86, *P* = .014). Conversely, the combined nursing strategy was associated with lower odds of gastric retention (OR = 0.31, *P* = .006). GCS score was negatively associated with gastric retention (OR = 0.81, *P* = .021), whereas age did not reach statistical significance (*P* = .112). These adjusted associations should not be interpreted as establishing an independent causal protective effect because residual confounding and nonrandom group allocation remain possible (Table 7).

**Table 7:** Logistic regression analysis.

Factor	β	OR	95% CI	*P*
Age	0.03	1.03	0.99–1.07	.112
Mechanical ventilation	0.82	2.27	1.01–5.08	.047
GCS score	−0.21	0.81	0.68–0.97	.021
Routine nursing care	1.05	2.86	1.23–6.64	.014
Combined monitoring (protective factor)	−1.18	0.31	0.13–0.72	.006

CI = confidence interval, GCS = Glasgow Coma Scale, OR = odds ratio.

## 4. Discussion

In this retrospective cohort, the combined nursing group had significantly lower observed incidences of abdominal distension, nausea and vomiting, and overall gastrointestinal intolerance than the control group. Gastric residual volume, time to achievement of nutritional targets, and gastrointestinal recovery indicators were also more favorable. Gastric retention occurred less frequently in the combined nursing group (15.0% vs 30.0%), although the unadjusted comparison did not reach statistical significance (*P* = .053). Hospital and ICU stays were shorter in the combined nursing group, but these findings represent associations and should not be attributed solely to the nursing model. Overall, the results support a potential clinical value of dynamic gastrointestinal assessment and individualized nursing adjustment while requiring cautious interpretation because of the observational design.

From a mechanistic perspective, neurosurgical patients often develop impaired gastrointestinal motility and delayed gastric emptying secondary to central nervous system injury through dysregulation of the brain–gut axis, thereby predisposing them to complications such as gastric retention and abdominal distension.^[16,17]^ Chapman et al confirmed that disordered gastric emptying is common among critically ill patients and constitutes an important pathological basis of enteral feeding intolerance.^[16]^ Reintam et al further noted that gastrointestinal motility disturbance is one of the major determinants of feeding failure and interruption of nutritional delivery.^[17]^ In the present study, dynamic monitoring of gastric residual volume and abdominal signs, together with timely adjustment of infusion strategies, effectively reduced gastrointestinal burden and thereby decreased the occurrence of related complications.

With regard to the effectiveness of nutritional therapy, the observation group achieved nutritional targets in a significantly shorter time, indicating that the combined intervention improved the efficiency of nutrient delivery. A systematic review by Elke et al showed that inadequate nutritional intake is closely associated with adverse outcomes in critically ill patients, whereas optimization of enteral nutrition strategies can significantly improve prognosis.^[18]^ Heyland et al likewise reported that early achievement of nutritional goals may reduce the risk of infection and mortality.^[19]^ Accordingly, adjustment of feeding strategies under the guidance of gastrointestinal function monitoring may contribute to more efficient nutritional support.

In terms of gastrointestinal recovery, the present study found that the recovery of bowel sounds and defecation occurred significantly earlier in the observation group, suggesting that the combined intervention facilitated restoration of gastrointestinal motility. Reintam et al proposed that gastrointestinal dysfunction is a common problem in critically ill patients and that its severity is closely related to prognosis.^[20]^ Subsequent studies have emphasized that dynamic assessment based on multiple indicators may support early identification of gastrointestinal dysfunction and guide timely intervention.^[21]^ The comprehensive assessment strategy adopted in this study is therefore in line with current trends in critical care nutrition management.

From a safety perspective, aspiration and reflux showed lower observed rates in the combined nursing group, although neither difference reached statistical significance, while enteral nutrition interruption was significantly less frequent. Aspiration is an important complication associated with enteral nutrition and is related to pulmonary infection and mortality risk.^[22]^ Bourgault et al reported an association of enteral feeding with microaspiration in critically ill adults.^[22]^ Poulard et al showed that changes in feeding management could improve continuity of nutritional support without increasing complications.^[23]^ The present findings therefore suggest a possible advantage in feeding continuity, while the nonsignificant aspiration and reflux results should not be overstated.

In addition, the observation group had significantly shorter hospital stays and ICU stays, indicating that this nursing model may improve overall clinical outcomes. Casaer et al reported that rational nutritional support strategies exert an important influence on recovery in critically ill patients.^[24]^ Arabi et al similarly demonstrated that optimization of nutritional management may reduce complication rates and promote rehabilitation.^[25]^ The current findings are consistent with these previous reports and further underscore the importance of optimizing nutrition-related nursing care.

In the analysis of risk factors, mechanical ventilation emerged as an important independent risk factor for gastric retention, which may be related to its effects on intra-abdominal pressure and gastrointestinal motility.^[26]^ Mentec et al also confirmed a significantly increased incidence of delayed gastric emptying in mechanically ventilated patients.^[26]^ At the same time, the GCS score was negatively associated with gastric retention, suggesting that more severe neurological impairment is accompanied by a greater risk of gastrointestinal dysfunction.^[27]^ Importantly, the association between the combined nursing strategy and lower odds of gastric retention should be interpreted as an adjusted association, not as evidence of an independent causal protective effect.

From the perspective of nursing practice, the present study highlights the importance of establishing a closed-loop management framework consisting of assessment, intervention, and reassessment. Compared with conventional fixed-process nursing care, dynamic monitoring places greater emphasis on individualized and precise intervention, which may contribute to improved quality of care.^[28]^ Compher et al reported that nurse-led nutritional management models can significantly enhance both the efficiency of nutritional implementation and patient tolerance.^[28]^

Several limitations should be acknowledged. First, this was a single-center retrospective study with a relatively small sample size, and selection bias cannot be excluded. Second, some variables and outcomes were derived from routine nursing documentation, which may introduce information and misclassification bias. Third, because the study was non-randomized, the way in which the combined nursing model was implemented (for example, by time period, ward/team, or clinical selection) could introduce temporal or selection bias; residual confounding remains possible even after multivariable adjustment. Fourth, no stratified analysis was performed according to specific neurosurgical disease categories. Fifth, if the criteria for nutritional tolerance and the nursing satisfaction instrument were locally developed rather than externally validated, measurement validity and generalizability may be limited. Finally, shorter hospital and ICU stays should be interpreted as associated findings rather than outcomes caused solely by the nursing model. Future multicenter prospective studies with larger samples, standardized outcome definitions, and validated patient-reported instruments are needed to confirm these findings.^[29,30]^

In summary, enteral nutrition nursing combined with gastrointestinal function monitoring was associated with better observed gastrointestinal tolerance, nutritional delivery, and gastrointestinal recovery in neurosurgical patients. The incidence of gastric retention was lower in the combined nursing group but did not differ significantly in the unadjusted comparison, while multivariable analysis suggested lower adjusted odds. The observed shorter hospital and ICU stays should be interpreted cautiously because residual confounding cannot be excluded. These findings support further prospective evaluation of this nursing strategy rather than definitive causal conclusions.

## Author contributions

**Conceptualization:** Huihui Lei, Man Wang, Ping Ke.

**Data curation:** Huihui Lei, Man Wang, Ping Ke.

**Formal analysis:** Huihui Lei, Man Wang, Ping Ke.

**Funding acquisition:** Ping Ke.

**Investigation:** Ping Ke.

**Writing – original draft:** Ping Ke.

**Writing – review & editing:** Ping Ke.
